# The Advanced Lung Cancer Inflammation Index: A Powerful and Independent Prognostic Factor in Metastatic Gastric Cancer

**DOI:** 10.3390/medicina61111949

**Published:** 2025-10-30

**Authors:** Tugay Avci, Mustafa Sahbazlar, Ferhat Ekinci, Atike Pinar Erdogan

**Affiliations:** Department of Medical Oncology, Faculty of Medicine, Manisa Celal Bayar University, Manisa 45030, Turkey; m_sahbazlar@hotmail.com (M.S.); drferhatekinci@hotmail.com (F.E.); dr_pinarcan@yahoo.com (A.P.E.)

**Keywords:** gastric cancer, metastatic, prognosis, advanced lung cancer inflammation index, ALI, survival, inflammatory marker

## Abstract

*Background and Objectives*: The Advanced Lung Cancer Inflammation Index (ALI), a composite marker integrating body mass index (BMI), serum albumin, and neutrophil-to-lymphocyte ratio (NLR), has demonstrated prognostic value in various cancers. However, its role in metastatic gastric cancer (mGC) remains unexplored. This study aimed to investigate the prognostic significance of the ALI in patients with mGC. *Materials and Methods*: This retrospective study included 206 patients with de novo or recurrent metastatic gastric cancer treated at Manisa Celal Bayar University between September 2009 and 2024. The ALI was calculated as “BMI × serum albumin (g/dL)/NLR”. A cut-off value of 20.8 was determined using Receiver Operating Characteristic (ROC) curve analysis. Patients were stratified into high (ALI > 20.8) and low (ALI ≤ 20.8) groups. Survival outcomes were evaluated using Kaplan–Meier analysis, and prognostic factors were examined with Cox regression models. *Results*: The median overall survival (OS) was significantly longer in the high-ALI group compared to the low-ALI group (11.4 months vs. 5.2 months, *p* < 0.001). The 1-year and 2-year OS rates were 42.9% and 11.3% for the high-ALI group, versus 11.6% and 2.1% for the low-ALI group, respectively. Multivariate Cox regression analysis confirmed that a low ALI was an independent predictor of poorer survival (HR: 2.07, 95% CI: 1.47–2.92, *p* < 0.001). The low-ALI group also had a higher prevalence of peritoneal metastases and a lower likelihood of receiving first- and second-line chemotherapy. *Conclusions*: The ALI is a strong and independent prognostic factor for survival in patients with metastatic gastric cancer. As an easily calculable and cost-effective index derived from routine parameters, it shows significant potential for risk stratification and treatment planning in clinical practice. Prospective, multi-centre studies are warranted to validate these findings.

## 1. Introduction

Gastric cancer is the fifth most common cancer worldwide and the fifth leading cause of cancer-related deaths [1]. Despite recent advances in treatment, it remains a significant cause of mortality. It is most commonly seen in people aged 65–74 and is more frequently diagnosed in men than in women [2]. In 2025, approximately 30,000 new cases of gastric cancer and approximately 11,000 gastric cancer-related deaths are expected in the United States, with gastric cancer patients accounting for 1.5% of newly diagnosed patients [2]. Although geographical variations exist, it is most commonly seen in East Asia, Eastern Europe, and South America [1]. Many factors, including genetic syndromes, dietary habits, and infectious processes such as Helicobacter pylori infection, play a role in the development of gastric cancer. The disease may be asymptomatic or present with dyspepsia, weight loss, persistent nausea–vomiting, and nonspecific symptoms (such as fatigue). Consequently, the majority of patients are diagnosed at the stage of regional lymph node involvement or metastasis. According to the Surveillance, Epidemiology, and End Results (SEER) 21 data, at the time of diagnosis, 31% of patients present with localised disease, 24% with locally advanced disease, and 36% with metastatic disease [2]. The 5-year survival rate is 76.5% for localised disease, 37.2% for locally advanced disease, and 7.5% for metastatic disease.

Although the primary treatment in the early stage is surgical resection, recurrence despite curative treatments remains a major problem. In the metastatic stage, the primary treatment is chemotherapy, and it has been shown that adding targeted agents and immunotherapies to chemotherapy prolongs survival [3,4].

Nutritional problems are common in gastric cancer patients, making it difficult for them to tolerate systemic treatments. This highlights the importance of identifying patients who require aggressive treatment at an early stage. There are numerous studies in the literature evaluating prognostic factors in gastric cancer [5,6].

Systemic inflammation associated with tumours plays a significant role in the progression and spread of cancer. This suggests that haematological and biochemical parameters indicative of systemic inflammation may be effective in determining prognosis. It is well known that systemic inflammation is a poor prognostic factor, typically associated with malnutrition, hypoalbuminaemia, weight loss, and cancer cachexia [7]. Research also supports this. For example, it has been established that the NLR, an indicator of systemic inflammation, is a strong predictor of the course of gastric cancer, as it is in many other types of cancer [8]. There are also studies evaluating the effect of malnutrition on prognosis in gastric cancer. The Controlling Nutritional Status (CONUT) score, which can be calculated from albumin concentration, lymphocyte count, and cholesterol concentration, is a tool used to assess the risk of malnutrition and has been shown to be useful in predicting clinical responses in gastric cancer [9]. In addition, studies have shown that the Glasgow Prognostic Score, platelet–lymphocyte ratio, and Naples Prognostic Score can predict survival in gastric cancer [10,11,12].

The Advanced Lung Cancer Inflammation Index (ALI), initially demonstrated as a prognostic marker in metastatic small cell lung cancer and later in gastrointestinal cancers such as colorectal cancer and pancreatic cancer, has been shown to be an independent prognostic factor in patients with operable gastric cancer based on its preoperative level [13,14,15]. The ALI index was first defined in 2013 by Jafri and colleagues and is formulated as “BMI × serum albumin (g/dL)/NLR” [13].

At the time this study was designed, there were no studies in the literature investigating whether this index was a prognostic marker in metastatic gastric cancer. The aim of this study was to determine whether the ALI is an independent prognostic factor in patients with metastatic gastric cancer and to evaluate the effect of this index on survival in conjunction with other clinical–pathological factors.

## 2. Materials and Methods

### 2.1. Study Design and Patient Population

This single-centre, retrospective cohort study was conducted at the Medical Oncology Outpatient Clinic of Manisa Celal Bayar University. Between September 2009 and September 2024, we screened the medical records of patients with histologically confirmed gastric cancer.

The study included 206 consecutive patients who met the following inclusion criteria: presence of de novo or recurrent metastatic disease, availability of complete baseline clinical, laboratory (complete blood count and serum albumin), and anthropometric (height and weight) data required for the calculation of the ALI at the time of metastatic diagnosis, and received at least one line of systemic therapy for metastatic disease at our clinic. Exclusion criteria were as follows: presence of a concurrent malignancy, incomplete medical records, or lost to follow-up shortly after diagnosis.

### 2.2. Data Collection and Variables

Patient data were meticulously extracted from the hospital’s electronic medical records and physical patient files. The collected variables included the following:

Demographics and Clinical Characteristics: Age at metastatic diagnosis, gender, body mass index (BMI) at baseline, Eastern Cooperative Oncology Group (ECOG) performance status, and comorbidities (hypertension, diabetes mellitus, chronic obstructive pulmonary disease, coronary artery disease).

Tumour-Related Factors:

- Histopathological subtype (adenocarcinoma, signet ring cell carcinoma, adenosquamous, medullary, poorly cohesive carcinoma).
- HER2 (human epidermal growth factor receptor 2) status (assessed by immunohistochemistry (IHC) and fluorescence in situ hybridisation (FISH) where applicable), categorised as negative, low (IHC 1+ or 2+/FISH-negative), or overexpressed (IHC 3+ or IHC 2+/FISH-positive).
- Microsatellite instability (MSI) status (microsatellite stable (MSS) or microsatellite instability high (MSI-H)).
- Metastatic status at presentation (de novo or recurrent).
- Sites of metastasis (liver, lung, bone, distant lymph nodes, peritoneum, other).

Treatment History:

- Prior gastrectomy and/or neoadjuvant/adjuvant therapy.
- Postoperative pathological TNM stage (for those who underwent surgery).
- Presence of lymphovascular and perineural invasion.
- First-line and subsequent lines of systemic therapy received in the metastatic setting.
- Use of targeted agents (trastuzumab).
- Receipt of palliative radiotherapy.

Laboratory Parameters (at Baseline): Serum albumin level (g/dL), absolute neutrophil count (×10^3^/µL), and absolute lymphocyte count (×10^3^/µL). The NLR was calculated as the absolute neutrophil count divided by the absolute lymphocyte count. BMI was calculated by dividing the patient’s weight in kilograms at the start of treatment by their height in metres squared. The ALI was calculated as BMI at the start of treatment × serum albumin (g/dL)/NLR.

### 2.3. Determination of the ALI Cut-Off Value

**Figure 1:**
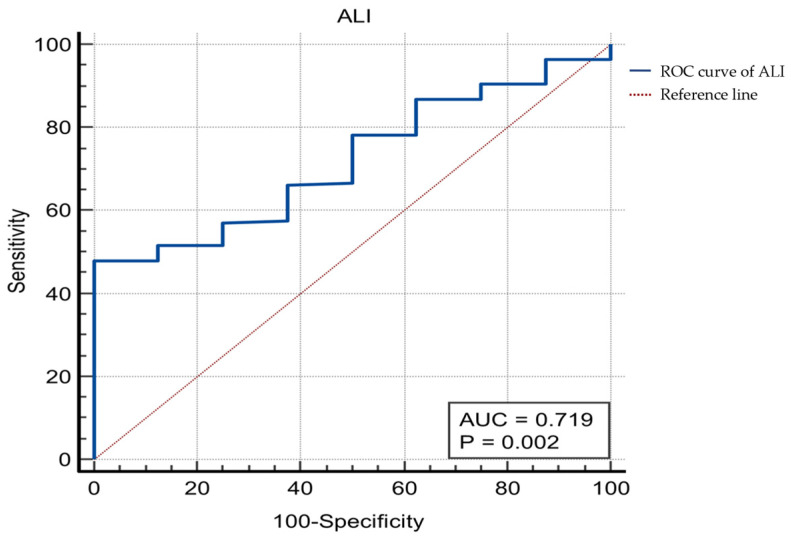
ROC curve analysis of ALI for overall survival.

To stratify patients into prognostically distinct groups, an optimal cut-off value for the ALI was determined using Receiver Operating Characteristic (ROC) curve analysis, with overall survival (OS) as the endpoint. The area under the curve (AUC) was 0.719 (95% CI: 0.652–0.779) (Figure 1). The Youden index identified an ALI ≤ 20.8 as the optimal cut-off value for predicting mortality, with a sensitivity of 47.98% and a specificity of 87.6%. Based on this, patients were dichotomised into two groups: a low-ALI group (ALI ≤ 20.8) and a high-ALI group (ALI > 20.8).

### 2.4. Statistical Analysis

SPSS 15.0 for Windows software was used for statistical analysis. Descriptive statistics were presented as counts and percentages for categorical variables and non-normally distributed variables were expressed as median (interquartile range), whereas normally distributed variables were presented as mean ± standard deviation. Proportions between groups were compared using the Chi-square test. Comparisons of numerical variables between groups were performed using the Mann–Whitney U test, as the condition of normal distribution was not met. Cut-off value analysis was performed using ROC curve analysis. Survival rates were examined using Kaplan–Meier analysis, and risk factors were examined using Cox regression analysis. The alpha significance level was set at *p* < 0.05.

## 3. Results

Of the patients included in the study, 145 (70.4%) were male and 61 (29.6%) were female. The median age of the patients was 64.5 (56–71) and was similar between the two groups. The median BMI of the patients was 23, and the BMI was statistically significantly higher in the high-ALI group (24.2 vs. 21.5, *p* ≤ 0.001). The most common comorbidities were hypertension (35.9%) and diabetes mellitus (22.3%), and comorbidities were similar in both arms. Although adenocarcinoma was the most common type in both groups, its incidence was higher in the high-ALI group. In the low-ALI group, the proportion of patients with poorly cohesive carcinoma and signet ring cell carcinoma (SRCC) was higher, and this was statistically significant when patients were compared according to subtype (*p* = 0.013). HER2 was examined in approximately half of the patients, and the FISH confirmed HER2+ patient rate was 5%. Peritoneal metastasis was statistically significantly higher in the low-ALI group (*p* = 0.024). The proportion of patients receiving first- and second-line chemotherapy was statistically significantly lower in the low-ALI group (*p* = 0.016 and *p* = 0.001, respectively). The other demographic and clinical characteristics of the patients were similar (Table 1).

The distribution of HER2, FISH, and MSI tests showed significant differences between calendar periods (*p* < 0.05 for all comparisons). As presented in Table 2, the frequency of testing increased progressively over time, reflecting the gradual standardisation and clinical integration of biomarker assessments between 2009 and 2024.

In 2009–2014, biomarker testing was relatively limited; approximately 74% of patients had not been tested for HER2 status, and FISH or MSI assessments had not been performed. In contrast, between 2015 and 2019, HER2 testing became more common (HER2 unknown: 58.5%), consistent with the February 2015 reimbursement approval of trastuzumab in Turkey, which led to more widespread use of HER2 testing in metastatic gastric cancer.

During the 2020–2024 period, the proportion of patients with unknown HER2 or MSI status decreased significantly (HER2 unknown: 13.9%; MSI unknown: 63.9%). This improvement is consistent with advances in laboratory infrastructure and studies demonstrating the efficacy of immunotherapies in these patients. Although nivolumab received reimbursement in our country for patients with metastatic gastric or gastro-oesophageal cancer in 2025, patients’ ability to access treatment through the courts prior to this date ensured that MSI was studied in more patients. Overall, the results show a clear temporal trend towards more systematic and comprehensive molecular profiling in metastatic gastric cancer. This indicates both an improvement in diagnostic capacity and adaptation to evolving treatment opportunities.

**Figure 2:**
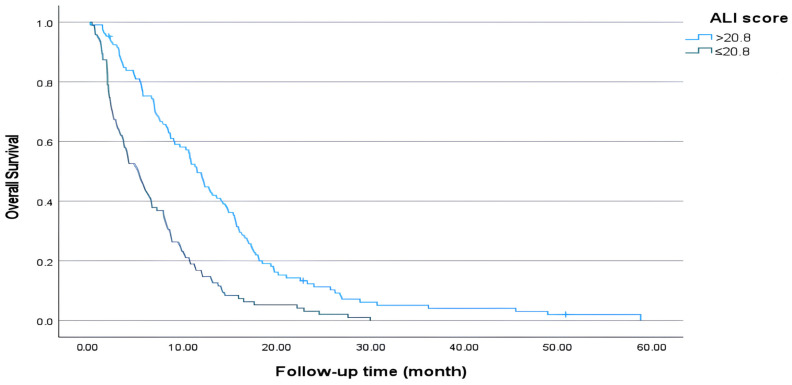
Overall survival (ALI ≤ 20.8 vs. >20.8).

When comparing OS, median survival was statistically significantly longer in the high-ALI group (median 11.4 months vs. 5.2 months, *p* < 0.001) (Figure 2). The proportion of patients alive at 6 months in the high-ALI group was approximately twofold higher than in the low-ALI group, and this proportion dramatically increased to approximately fivefold by 2 years (Table 3).

**Figure 3:**
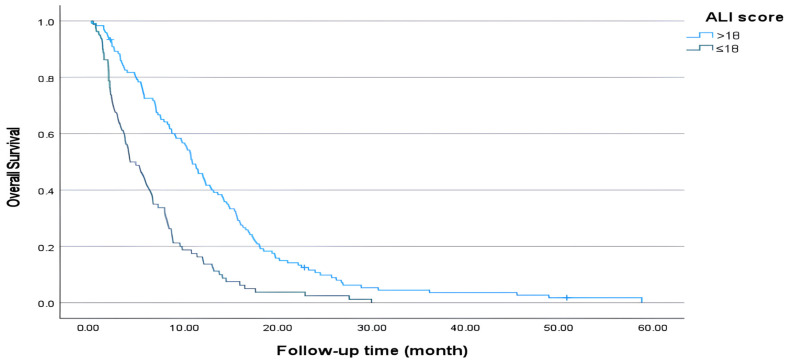
Overall survival (ALI ≤ 18 vs. >18).

In our study, patients with an ALI > 20.8 were considered to have a high ALI, whereas in some studies, an ALI > 18 was considered to be a high ALI. When patient survival was re-evaluated in this manner, median survival was statistically significantly longer in those with an ALI > 18 compared to those with an ALI ≤ 18 (10.8 months vs. 4.1 months, *p* < 0.001) (Figure 3). At two years, overall survival was about fourfold higher in the high-ALI group compared with the low-ALI group (respectively 9.9% vs. 2.5%) (Table 4).

When examining risk factors that could affect patient survival, the single Cox regression analysis (Table 5) showed that the ALI was the strongest prognostic factor, with patients in the low-ALI group having a 2.25-fold higher risk of death compared to patients in the high-ALI group (*p* < 0.001, HR = 2.24 (95% CI: 1.68–3.0)). Each unit increase in the ALI score was also associated with an approximately 1.7% decrease in the risk of death (*p* < 0.001, HR = 0.98 (95% CI: 0.97–0.99)). The presence of bone metastases increased the risk of mortality by approximately 60% (*p* = 0.018, HR = 1.59 (95% CI: 1.08–2.35)). An increase in BMI was associated with a minimal but significant reduction in risk (*p* = 0.036, HR = 0.96 (95% CI: 0.93–0.99)). Receiving systemic chemotherapy was associated with a reduction in mortality risk of over 65%, while those receiving second-line chemotherapy also experienced a reduction in mortality risk of approximately 62%, which was statistically significant. Patients receiving palliative radiotherapy during the metastatic phase also experienced a 44% reduction in mortality. Similarly, each unit increase in the number of treatment lines was associated with an approximately 42% reduction in the risk of death. This indicated that the patient responded to treatment and was able to benefit from more treatment options, which led to a significant reduction in mortality.

When factors that could affect mortality were evaluated using multiple Cox regression analysis, the mortality risk was approximately two times higher in the low-ALI group (*p* < 0.001, HR = 2.07 (95% CI: 1.47–2.92) (Table 6). Regarding treatment-related factors, radiotherapy was associated with a significant reduction in mortality risk (HR = 0.64, 95% CI: 0.42–0.99, *p* = 0.048), suggesting that local control may translate into a survival advantage in selected metastatic cases. Similarly, patients who received second-line chemotherapy had a notably lower risk of death (HR = 0.47, 95% CI: 0.26–0.83, *p* = 0.009), emphasising the importance of maintaining treatment continuity beyond first-line therapy. Patients with poor performance status (ECOG 2) exhibited a significantly lower risk of death compared with those with ECOG 0 (HR = 0.45, 95% CI: 0.24–0.83, *p* = 0.011). This somewhat unexpected result may be attributed to selection bias, as patients with ECOG 2 who survived long enough to receive treatment might represent a subgroup with better tolerance or favourable disease biology. Conversely, age, sex, comorbidities (hypertension, diabetes mellitus, COPD, coronary artery disease), metastatic sites (liver, lung, bone, lymph nodes, peritoneum), and year of diagnosis were not significantly associated with survival (all *p* > 0.05). Overall, these results highlight that systemic inflammation and nutritional status (as reflected by the ALI), along with performance capacity and access to subsequent therapies, are key determinants of prognosis in metastatic gastric cancer.

## 4. Discussion

This retrospective study demonstrates that the ALI at the start of treatment is a strong and independent prognostic indicator in patients with metastatic gastric cancer. Our findings indicate that overall survival is significantly shorter in patients with low ALI values and that this index retains its independent effect in predicting survival irrespective of other clinical–pathological factors. This suggests that the ALI could potentially be used as a tool for risk stratification in metastatic gastric cancer and for identifying patients with a poorer prognosis.

The ALI is an index that assesses the patient’s nutritional status and systemic inflammatory response based on parameters, such as BMI, serum albumin, and NLR. The detection of lower albumin and BMI in the low-ALI group in our study suggests that this group reflects a pronounced cachectic and pro-inflammatory state. Cancer cachexia and systemic inflammation are known to be associated with tumour progression, suppression of the immune response, and reduced survival [7]. Therefore, the ALI may gain its prognostic power by integrating these pathophysiological processes into a single parameter.

The prognostic value of the ALI has previously been demonstrated in lung, pancreatic, and colorectal cancers; in gastric cancer, it has been studied in surgically treated, locally advanced patients [13,14]. Reviewing these studies, a retrospective analysis of 459 gastric cancer patients showed that a low preoperative ALI was associated with poorer overall survival. Five-year OS was 85.5% in the low-ALI group, whereas this rate was 93.8% in the high-ALI group and was statistically significant (*p* = 0.01) [15]. Again, in the same study, five-year relapse-free survival was 82.1% in the low-ALI group, whereas it was 91.8% in the high-ALI group (*p* = 0.02). Another retrospective study involving 620 patients found that both OS and DFS were statistically significantly lower in the low-ALI group, and multivariate analysis revealed that the ALI was an independent prognostic factor for OS [16]. Similarly, a study involving 1.657 patients showed that a low ALI was associated with poorer OS and cancer-specific survival (*p* < 0.001 and *p* = 0.001, respectively). Multivariate analysis revealed that a low ALI was associated with a 1.55-fold increase in the risk of death (*p* = 0.01). Our study contributes to the literature as it is the first to demonstrate a similar relationship between survival and the ALI in the metastatic stage.

The higher incidence of peritoneal metastasis in the low-ALI group in our study suggests that these patients may have a more biologically aggressive tumour phenotype. Furthermore, the significantly lower rates of first- and second-line chemotherapy in the low-ALI group indicate that these patients have limited treatment tolerance and cannot receive intensive treatment in the early stages. This finding suggests that the ALI can be used not only as a prognostic tool but also as a predictive tool in treatment planning.

In this study, multivariable Cox regression analysis identified several independent prognostic factors affecting overall survival in patients with metastatic gastric cancer. Among these, ECOG performance status, a high ALI, radiotherapy, and second-line chemotherapy were found to be significant determinants of survival outcomes.

Our findings confirmed the prognostic relevance of systemic inflammation and nutritional status, as reflected by the ALI. Patients with a low ALI experienced approximately twofold higher mortality risk compared with those with a high ALI, underscoring the strong influence of inflammation-based markers on cancer prognosis. This result is consistent with previous studies reporting that a low ALI is associated with poor nutritional reserves, higher systemic inflammatory burden, and ultimately worse clinical outcomes in gastrointestinal malignancies [14,15].

The relationship between ECOG performance status and survival was also statistically significant. Interestingly, patients with ECOG 2 appeared to have a lower hazard ratio compared with those with ECOG 0. This unexpected finding may reflect a selection bias, as patients with ECOG 2 who were included in the analysis might represent a subgroup with adequate physiological reserves or better response to therapy despite a nominally poor performance status. It also highlights the heterogeneity of functional assessment in real-world clinical practice.

Palliative radiotherapy in patients with metastatic gastric cancer is primarily used to achieve haemostasis in patients with gastric bleeding. However, a study conducted by Hingaroni et al. demonstrated that palliative radiotherapy administered alongside systemic treatment in metastatic gastric cancer significantly improves survival [17]. In this study, the median survival in patients receiving palliative radiotherapy was 23 months. Similar to this study, our study also found that radiotherapy has a positive effect on survival. This indicates that local control of the disease may contribute to systemic benefits or that patients with oligometastatic or symptomatic disease who may benefit from such interventions are carefully selected. Additionally, the risk of mortality was significantly reduced in patients receiving second-line chemotherapy. This underscores the importance of continuing treatment beyond first-line therapy when clinically appropriate. On the other hand, variables such as age, sex, comorbidities (hypertension, diabetes mellitus, COPD, coronary artery disease), metastatic sites, and calendar year of diagnosis did not show significant associations with survival. This suggests that biological and treatment-related factors, rather than demographic characteristics, play a more decisive role in patient prognosis in the metastatic setting.

Limitations of our study include its retrospective design, its single-centre nature, and the inability to evaluate certain biomarkers (e.g., MSI, HER2) in all patients. However, the large patient number and long follow-up period are among the strengths of the study.

## 5. Conclusions

In conclusion, our study demonstrates that the ALI is a strong and independent prognostic factor in patients with metastatic gastric cancer. Patients with a low ALI had significantly shorter overall survival compared to those with a high ALI. A low ALI was independently associated with a twofold increased risk of mortality. Moreover, the low-ALI group exhibited a higher incidence of peritoneal metastasis and received systemic therapy less frequently, suggesting poorer treatment tolerance and more aggressive disease biology. Given its simplicity, low cost, and reliance on routinely available parameters, the ALI can serve as a practical biomarker for risk stratification and treatment planning in metastatic gastric cancer. Prospective, multi-centre studies are warranted to validate these findings and further define its role in clinical decision-making.

## Figures and Tables

**Table 1:** Demographic and clinical characteristics of patients.

		Total (n = 206)	ALI Group		
			>20.8 (n = 111)	≤20.8 (n = 95)	
		n (%)	n (%)	n (%)	*p*
Gender	Male	145 (70.4)	80 (72.1)	65 (68.4)	0.567
	Female	61 (29.6)	31 (27.9)	30 (31.6)	
Age median (min-max)		64.5 (56–71)	65 (58–71)	63 (55–70)	0.175
BMI median (min-max)		23 (20.6–26.3)	24.2 (21.2–27.9)	21.5 (20–24.2)	**<0.001**
Comorbidities	HT	74 (35.9)	42 (37.8)	32 (33.7)	0.536
	DM	46 (22.3)	28 (25.2)	18 (18.9)	0.281
	COPD	23 (11.2)	13 (11.7)	10 (10.5)	0.788
	CAD	37 (18)	25 (22.5)	12 (12.6)	0.065
ECOG	0	86 (41.7)	44 (39.6)	42 (44.2)	0.595
	1	88 (42.7)	51 (45.9)	37 (38.9)	
	2	32 (15.5)	16 (14.4)	16 (16.8)	
Pathological subtype	Adenocarcinoma	166 (80.6)	98 (88.3)	68 (71.6)	**0.013**
	SRCC	26 (12.6)	10 (9.0)	16 (16.8)	
	Adenosquamous	2 (1.0)	0 (0)	2 (2.1)	
	Medullary	1 (0.5)	0 (0)	1 (1.1)	
	Poorly cohesive carcinoma	11 (5.3)	3 (2.7)	8 (8.4)	
HER2 IHC	Negative	63 (30.6)	37 (33.3)	26 (27.4)	0.492
	1+	15 (7.3)	10 (9.0)	5 (5.3)	
	2+	6 (2.9)	2 (1.8)	4 (4.2)	
	3	12 (5.8)	5 (4.5)	7 (7.4)	
	Unknown	110 (53.4)	57 (51.4)	53 (55.8)	
FISH	Negative	15 (7.3)	7 (6.39)	8 (8.4)	0.698
	Positive	11 (5.3)	5 (4.5)	6 (6.3)	
	Unknown	180 (87.4)	99 (89.2)	81 (85.39	
HER2 status	Low	22 (10.7)	12 (10.8)	10 (10.5)	0.678
	Overexpression	11 (5.3)	5 (4.5)	6 (6.3)	
	Negative	60 (29.1)	36 (32.4)	24 (25.3)	
	Unknown	113 (54.9)	58 (52.3)	55 (57.9)	
MSI	Negative—MSS	20 (9.7)	8 (7.2)	12 (12.6)	0.145
	Positive—MSH	3 (1.5)	3 (2.7)	0 (0.0)	
	Unknown	183 (88.8)	100 (90.1)	83 (87.4)	
Operation		72 (35.0)	40 (36.0)	32 (33.7)	0.724
Metastasis status	De novo metastatic	157 (76.2)	79 (71.2)	78 (82.1)	0.066
	Recurrent metastatic	49 (23.8)	32 (28.8)	17 (17.9)	
Neoadjuvant therapy	Not received	44 (89.8)	27 (84.4)	17 (100)	0.259
	FLOT	3 (6.1)	3 (9.4)	0 (0.0)	
	Other	2 (4.1)	2 (6.3)	0 (0.0)	
Postoperative lymph node stage	N0	7 (10.1)	3 (7.9)	4 (12.9)	0.442
	N1	12 (17.4)	9 (23.7)	3 (9.7)	
	N2	11 (15.9)	5 (13.2)	6 (19.4)	
	N3	39 (56.5)	21 (55.3)	18 (58.1)	
Postoperative T stage	T2	5 (7.2)	3 (7.9)	2 (6.5)	0.597
	T3	14 (20.3)	6 (15.8)	8 (25.8)	
	T4	50 (72.5)	29 (76.3)	21 (67.7)	
Postoperative M stage	M0	44 (63.8)	28 (73.7)	16 (51.6)	0.058
	M1	25 (36.2)	10 (26.3)	15 (48.4)	
Lymphovascular invasion		58 (84.1)	32 (84.2)	26 (83.9)	1000
Perineural invasion		57 (82.6)	33 (86.8)	24 (77.4)	0.304
Metastasis site	Liver	83 (40.3)	45 (40.5)	38 (40.0)	0.937
	Lung	39 (18.9)	23 (20.7)	16 (16.8)	0.479
	Bone	32 (15.5)	17 (15.3)	15 (15.8)	0.925
	Distant LN	176 (85.4)	93 (83.8)	83 (87.4)	0.467
	Peritoneum	91 (44.2)	41 (36.9)	50 (52.6)	**0.024**
	Other	58 (28.2)	32 (28.8)	26 (27.4)	0.573
First-line treatment		174 (84.5)	100 (90.1)	74 (77.9)	**0.016**
Trastuzumab		8 (3.9)	5 (4.5)	3 (3.2)	0.618
Radiotherapy		41 (19.9)	26 (23.4)	15 (15.8)	0.171
Second-line treatment		80 (39.0)	55 (50.0)	25 (26.3)	**<0.001**
Progression status		198 (98.0)	105 (97.2)	93 (98.9)	0.625

BMI: body mass index, ECOG: Eastern Cooperative Oncology Group, HT: hypertension, DM: diabetes mellitus, COPD: chronic obstructive lung disease, CAD: coronary artery disease, HER2: human epidermal growth factor receptor 2, IHC: immunohistochemistry, FISH: fluorescence in situ hybridisation, MSI: microsatellite instability, MSS: microsatellite stable, MSH: microsatellite instability high, FLOT: fluorouracil–leucovorin–oxaliplatin–docetaxel protocol, LN: lymph node, SRCC: signet ring cell carcinoma.

**Table 2:** Changes in the frequency and distribution of HER2, FISH, and MSI testing over calendar periods (2009–2024).

	2009–2014	2015–2019	2020–2024	*p*
HER2	25.7%	41.5%	86.1%	***p*** ** < 0.001**
FISH	0%	14.1%	19.4%	***p*** ** = 0.024**
MSI	0%	7.4%	36.1%	**<0.001**

**Table 3:** Comparison of overall survival among patients (ALI ≤ 20.8 vs. >20.8).

ALI	mOS (m)	OS %				
		6 m	1 yr	2 yr	3 yr	
**≤20.8**	5.2	37.9	11.6	2.1		***p* < 0.001**
**>** **20.8**	11.4	69.6	42.9	11.3	4.1	
**Overall**	8.1	54.5	29.0	6.9	2.1	

ALI: the Advanced Lung Cancer Inflammation Index, OS: overall survival, mOS: median overall survival, m: month, yr: year.

**Table 4:** Comparison of overall survival among patients (ALI ≤ 18 vs. >18).

ALI	mOS (m)	OS %				
		6 m	1 yr	2 yr	3 yr	
≤18	4.1	34.6	12.3	2.5		***p* < 0.001**
>18	10.8	68.1	40.4	9.9	3.6	
Overall	8.1	54.5	29.0	6.9	2.1	

ALI: the Advanced Lung Cancer Inflammation Index, OS: overall survival, mOS: median overall survival, m: month, yr: year.

**Table 5:** Univariate Cox regression analysis for risk factors of death.

		*p*	HR	95% CI	
				Min	Max
Age at diagnosis		0.964	1.000	0.988	1.012
Gender	Female *	0.175	1.243	0.908	1.701
	Male				
BMI		**0.036**	0.966	0.936	0.998
HT		0.920	1.015	0.757	1.361
DM		0.092	0.748	0.534	1.048
COPD		0.721	1.083	0.700	1.677
CAD		0.480	1.140	0.793	1.639
ECOG	0 *	0.925			
	1	0.697	0.941	0.694	1.277
	2	0.832	0.955	0.623	1.464
Pathological subtype	Adenocarcinoma *	0.345			
	SRCC	0.577	1.128	0.738	1.724
	Adenosquamous	0.482	1.651	0.408	6.689
	Medullary	0.492	1.998	0.278	14.38
	Poorly cohesive carcinoma	0.059	1.862	0.976	3.553
HER2 IHC	Negative *	0.999			
	1+	0.930	1.026	0.581	1.811
	2+	0.810	0.902	0.388	2.096
	3+	0.940	0.976	0.523	1.822
	Unknown	0.951	0.990	0.718	1.364
FISH	Negative *	0.973			
	Positive	0.820	1.095	0.502	2.388
	Unknown	0.855	1.051	0.619	1.783
HER2 status	Low *	0.981			
	Overexpressed	0.822	1.087	0.527	2.244
	Negative	0.747	1.085	0.662	1.777
	Unknown	0.921	1.024	0.646	1.621
MSI	Negative—MSS *	0.796			
	Positive—MSH	0.500	0.658	0.194	2.225
	Unknown	0.773	0.932	0.580	1.500
Operation		0.440	1.123	0.837	1.508
Metastasis status	De novo metastatic *	0.726	0.942	0.673	1.318
	Recurrent metastatic				
Neoadjuvant chemotherapy	Not received *	0.599			
	FLOT	0.317	2.093	0.492	8.908
	Other	0.843	1.156	0.275	4.850
Postoperative lymph node stage	N0 *	0.914			
	N1	0.842	0.901	0.323	2.511
	N2	0.959	0.974	0.352	2.694
	N3	0.769	1.140	0.476	2.730
Postoperative T stage	T2 *	**0.026**			
	T3	**0.028**	3.670	1.147	11.74
	T4	0.339	1.655	0.590	4.644
Postoperative M stage	M0 *	0.992	0.998	0.600	1.659
	M1				
Lymphovascular invasion		0.829	0.925	0.454	1.883
Perineural invasion		0.758	1.113	0.563	2.203
ALI score		**<0.001**	0.983	0.977	0.990
ALI group	>20.8 *	**<0.001**	2.246	1.681	3.000
	≤20.8				
Liver metastasis		0.252	1.182	0.888	1.573
Lung metastasis		0.186	0.782	0.543	1.126
Bone metastasis		**0.018**	1.598	1.084	2.354
Distant LN metastasis		0.275	0.796	0.529	1.198
Peritoneal metastasis		0.117	1.256	0.944	1.669
First-line chemotherapy		**<0.001**	0.345	0.233	0.509
Trastuzumab		0.751	0.892	0.439	1.812
Radiotherapy		**0.002**	0.557	0.387	0.802
Second-line chemotherapy		**<0.001**	0.376	0.279	0.506
Number of treatment lines		**<0.001**	0.582	0.494	0.686
Progression		0.676	0.657	0.091	4.712

BMI: Body mass index, ECOG: Eastern Cooperative Oncology Group, HT: hypertension, DM: diabetes mellitus, COPD: chronic obstructive lung disease, CAD: coronary artery disease, HER2: human epidermal growth factor receptor 2, IHC: immunohistochemistry, FISH: fluorescence in situ hybridisation, MSI: microsatellite instability, MSS: microsatellite stable, MSH: microsatellite instability high, FLOT: fluorouracil–leucovorin–oxaliplatin–docetaxel protocol, LN: lymph node, SRCC: signet ring cell carcinoma, ALI: the Advanced Lung Cancer Inflammation Index, *: reference.

**Table 6:** Multivariate Cox regression analysis for risk factors of death.

		*p*	HR	95% CI	
				Min	Max
Age		0.959	1.000	0.982	1.018
Gender	Female *	0.058	1.472	0.986	2.197
	Male				
HT		0.432	1.176	0.785	1.763
DM		0.189	0.751	0.489	1.152
COPD		0.659	0.888	0.524	1.505
CAD		0.103	1.448	0.928	2.258
ECOG	0 *	0.033			
	1	0.379	0.828	0.543	1.261
	2	**0.011**	0.450	0.244	0.832
ALI group	>20.8 *	**0.001**	2.077	1.477	2.920
	≤20.8				
Metastasis status	De novo metastatic	0.599	1.127	0.721	1.762
	Recurrent metastatic				
Liver metastasis		0.129	1.291	0.928	1.796
Lung metastasis		0.468	0.860	0.572	1.292
Bone metastasis		0.233	1.309	0.841	2.037
Distant LN metastasis		0.442	0.822	0.499	1.355
Peritoneal metastasis		0.543	1.107	0.797	1.537
First-line chemotherapy		0.091	0.597	0.329	1.086
Trastuzumab		0.748	1.140	0.512	2.536
Radiotherapy		**0.048**	0.644	0.417	0.996
Second-line chemotherapy		**0.009**	0.465	0.261	0.827
Number of treatment lines		0.207	0.818	0.599	1.117
Year of diagnosis	2009–2014 *	0.480			
	2015–2019	0.596	0.886	0.568	1.384
	2020–2024	0.610	1.170	0.641	2.135

ECOG: Eastern Cooperative Oncology Group. HT: hypertension. DM: diabetes mellitus. COPD: chronic obstructive lung disease. CAD: coronary artery disease. ALI: the Advanced Lung Cancer Inflammation Index. *: reference.

## Data Availability

The raw data supporting the conclusions of this article will be made available by the authors upon request.
